# Not sick enough to worry? "Influenza-like" symptoms and work-related behavior among healthcare workers and other professionals: Results of a global survey

**DOI:** 10.1371/journal.pone.0232168

**Published:** 2020-05-13

**Authors:** Ermira Tartari, Katja Saris, Nikki Kenters, Kalisvar Marimuthu, Andreas Widmer, Peter Collignon, Vincent C. C. Cheng, Shuk C. Wong, Thomas Gottlieb, Paul A. Tambyah, Eli Perencevich, Benedetta Allegranzi, Angela Dramowski, Michael B. Edmond, Andreas Voss, on behalf of the International Society of Antimicrobial Chemotherapy Infection and Prevention Control (ISAC-IPC) Working Group

**Affiliations:** 1 Infection Control Programme and WHO Collaborating Centre on Patient Safety, Geneva University Hospitals and University of Geneva Faculty of Medicine, Geneva, Switzerland; 2 Institute of Global Health, Faculty of Medicine, University of Geneva, Geneva, Switzerland; 3 Faculty of Health Sciences, University of Malta, Msida, Malta; 4 Department of Medical Microbiology and Infectious Diseases, Canisius-Wilhelmina Hospital (CWZ), Nijmegen, The Netherlands; 5 Department of Medical Microbiology, Radboudumc, Nijmegen, The Netherlands; 6 REshape Center for Innovation, Radboudumc, Nijmegen, The Netherlands; 7 Department of Infectious Diseases, Tan Tock Seng Hospital, Singapore, Singapore; 8 National Centre for Infectious Diseases, Singapore, Singapore; 9 Yong Loo Lin School of Medicine, National University of Singapore, Singapore, Singapore; 10 University of Basel Hospitals and Clinics, Basel, Switzerland; 11 Medical School, The Australian National University, Canberra, ACT, Australia; 12 Department of Microbiology, Queen Mary Hospital, The University of Hong Kong, Hong Kong SAR, China; 13 Infection Control Team, Queen Mary Hospital, Hong Kong West Cluster, Hospital Authority, Hong Kong SAR, China; 14 Department of Microbiology and Infectious Diseases Concord Repatriation General Hospital, Sydney, NSW, Australia; 15 Department of Medicine, National University of Singapore, Singapore, Singapore; 16 Divisions of General Internal Medicine and Infectious Diseases, University of Iowa Carver College of Medicine, Iowa City, IA, United States of Amrerica; 17 Infection Prevention and Control Technical and Clinical Hub, Department of Integrated Health Services, World Health Organization, Geneva, Switzerland; 18 Department of Paediatrics and Child Health, Division of Paediatric Infectious Diseases, Stellenbosch University, Cape Town, South Africa; 19 University of Iowa Hospitals and Clinics, Iowa City, IA, United States of America; Ben-Gurion University of the Negev, UNITED STATES

## Abstract

**Background:**

Healthcare workers (HCWs) and non-HCWs may contribute to the transmission of influenza-like illness (ILI) to colleagues and susceptible patients by working while sick (presenteeism). The present study aimed to explore the views and behavior of HCWs and non-HCWs towards the phenomenon of working while experiencing ILI.

**Methods:**

The study was a cross-sectional online survey conducted between October 2018 and January 2019 to explore sickness presenteeism and the behaviour of HCWs and non-HCWs when experiencing ILI. The survey questionnaire was distributed to the members and international networks of the International Society of Antimicrobial Chemotherapy (ISAC) Infection Prevention and Control (IPC) Working Group, as well as via social media platforms, including LinkedIn, Twitter and IPC Blog.

**Results:**

In total, 533 respondents from 49 countries participated (Europe 69.2%, Asia-Pacific 19.1%, the Americas 10.9%, and Africa 0.8%) representing 249 HCWs (46.7%) and 284 non-HCWs (53.2%). Overall, 312 (58.5%; 95% confidence interval [CI], 56.2–64.6) would continue to work when sick with ILI, with no variation between the two categories. Sixty-seven (26.9%) HCWs and forty-six (16.2%) non-HCWs would work with fever alone (p<0 .01) Most HCWs (89.2–99.2%) and non-HCWs (80%-96.5%) would work with “minor” ILI symptoms, such as sore throat, sinus cold, fatigue, sneezing, runny nose, mild cough and reduced appetite.

**Conclusion:**

A future strategy to successfully prevent the transmission of ILI in healthcare settings should address sick-leave policy management, in addition to encouraging the uptake of influenza vaccine.

## Introduction

As the world watches the current COVID-19 pandemic unfold, the global public threat of severe respiratory infectious disease has created an unprecedented challenge [1, 2]. Attention has been drawn to the transmission of respiratory disease between patients and healthcare workers (HCWs), thus highlighting the need to prioritize infection prevention that ensures safe delivery of patient care [3]. Consequently, there is a growing interest in the phenomenon of sickness presenteeism, defined as attending work through illness, which has now been perceived as a challenge across several sectors, with the highest rates reported in human service organization (care, welfare and education) [4]. It is of particular concern in healthcare where there is a potentially serious public health impact due to the risk of cross-infection [3] to susceptible populations and excess morbidity and mortality [5, 6]. Sickness presenteeism may compromise patient safety and the quality of healthcare delivered [7] and is associated with societal costs through productivity loss [8], impaired judgement [9] and an increased risk of medical errors [10]. An association between mental exhaustion and susceptibility to the common cold has also been reported [11]. The behavior of working while ill has important implications for the workers themselves, with reported associations of sickness presenteeism and depression, musculoskeletal injury [4] and coronary heart disease [12], and increased sickness absenteeism [13].

HCWs presenting to work while ill can be significant contributors to the transmission of influenza to patients in healthcare facilities [14, 15]. Indeed, the prevalence of sickness presenteeism reported in cross-sectional surveys of different occupational roles varies between 40% and 90% [14–17]. Numerous outbreaks of healthcare-associated influenza or influenza-like illness (ILI) involving HCWs as the primary reservoir for transmission have been described in both acute and long-term healthcare facilities, including oncology and geriatric units, thus highlighting infection prevention and control challenges to be met [18–21]. It has been reported that HCWs continue to work while experiencing ILI even when caring for immunocompromised transplant recipients [22] and a recent cohort study [23] reported a high prevalence of presenteeism (14–23%) in HCWs with laboratory-positive influenza infection.

A variety of organizational, cultural and individual factors are cited as the most common reasons for fostering presenteeism. The workplace culture and safety climate play an important role. This includes policies, procedures and inherent constraints, such as job insecurity, the societal insurance system and limited replaceability, which may be a consequence of understaffing and where an insufficient number of allocated sick leave days leads to the perception that the HCW is unable to afford time off [24]. For example, Ridgway et al [24] found that a hospital’s sickness absence policy prohibited febrile HCWs from working, but not those with respiratory symptoms. Another reason cited for presenteeism is related to individual factors, such as personal financial consequences. Employees felt they were not unwell enough to miss work, not thinking they could be vectors for transmission, and unsure of what constitutes “too sick to work” (the case for those who were asymptomatic or with very mild symptoms) [15, 24]. Other concerns included having a professional obligation, placing a burden on colleagues, fear of ostracism by managers and coworkers, concern about continuity of care and a prevalent cultural norm to come to work unless very ill, thus underlining the ambiguity around what constitutes “too sick to work” [24–26].

Despite progress on developing resilient health systems to prevent healthcare-associated infections and calls to increase the uptake of the annual influenza vaccine as a primary preventive measure, [27] insufficient emphasis has been given to the prevention or reduction of sickness presenteeism within organizational structures [15]. Considering its importance as a public health concern, we sought to describe the behavior, practices and perceptions of HCWs and other professionals on working while sick with ILI, with the aim to identify areas for future improvement and to encourage organizations to have comprehensive ILI prevention and control strategies in place.

## Methods

A web-based questionnaire was developed and to collect responses anonymously.

The study was approved by the medical research ethical committee of Arnhem-Nijmegen (The Netherlands) (NL2018-4258). All participants received information about the purpose of the study, the survey completion time (5–10 minutes), and assurance of the confidentiality and anonymity of the survey responses. Submission of responses via the online platform indicated agreement to participate in the survey.

### Study design, participants and recruitment

In October 2018, the International Society of Antimicrobial Chemotherapy (ISAC) Working Group for Infection Prevention and Control (IPC) launched a cross-sectional survey of ILI sickness presenteeism. The IPC Working Group supports international cooperation with regards to education, research and guidelines in IPC in healthcare settings worldwide.

ISAC has affiliated members and experts from 57 countries and 65 regions in all six continents worldwide. Participation in the survey was based mainly on interest rather than on a systematic sampling process. Participants completed a web-based survey tool, which was generated using Survey Monkey® (SurveyMonkey®, San Mateo, CA, USA). The survey was available online between 25 October 2018 and 11 January 2019. The link to the survey was distributed through ISAC and IPC Working Group members and networks, and also via social platforms, including LinkedIn, Twitter, and IPC Blog, with the request to distribute further to the networks in their countries/continents. Both HCWs and other professionals (public) were invited to participate on a voluntary basis. No financial incentives were offered for participation.

### Survey instrument

After reviewing the literature, a team of investigators, including infectious disease and IPC experts developed the survey instrument (S1 File). The survey was distributed among IPC experts within the ISAC-IPC Working Group to test for understandability and clarity, especially in countries where English was not the primary language, and to reach consensus on the content. The platform was piloted to evaluate usability and any technical failures. The survey was piloted in 10 countries in all six continents. The final survey was five pages in length and comprised 17 items.

We collected data on gender, age, geographical location, educational status and employment. The items of the questionnaire were classified into the following topics: 1) knowledge questions where participants were asked to select the most important ILI symptoms; 2) ILI-related behavior: symptoms reported for coming into work when sick, symptoms that would keep someone at home and away from work when sick, and symptoms present when returning to work after having been unwell with an ILI; 3) attitudes regarding avoiding people at work with ILI-related symptoms and family or friends with ILI-related symptoms; 4) willingness to receive the annual influenza vaccine; and 5) ILI-related sickness in the past two years. The case definition for ILI was subjective, rather than an epidemiological case definition, with a number of symptoms categorized into "major" +/- "minor". The survey included dichotomous (yes/no) and closed-ended questions. Questions regarding ILI symptoms allowed participants to select more than one identifying feature, while questions that addressed sickness presenteeism behavior and attitudes allowed for only one type of response. Every possible answer to a question was allocated a numerical score on the survey platform.

### Statistical analysis

R version 3.5.1 was used for the statistical analysis (R Core Team. R: A language and environment for statistical computing. R Foundation for Statistical Computing, Vienna, Austria; 2017; https://www.R-project.org/).Responses were organized into categories for the purpose of descriptive statistics. Summary statistics were calculated for the demographic variables and questions on ILI-related symptoms. Differences between groups were tested using the chi-square test for categorical variables. A two-sided p<0.05 was considered significant.

## Results

### Characteristics of participants

A total of 571 survey responses was submitted. Among these, 14 duplicates were excluded and only 533 were sufficiently complete to be included in the analysis. Table 1 summarizes the sociodemographic characteristics of participants.

**Table 1:** Demographics of participants.

Demographics	No. (%) of participants (n = 533)
**Gender**	
Female	376 (70.5)
Male	157 (29.5)
**Age category (years)**	
18–29	62 (11.6)
30–49	261 (49.0)
50+	210 (39.4)
**Level of education**	
Primary	7 (1.3)
Secondary	85 (15.9)
Higher	441 (82.7)
**Region**	
Northern Europe	278 (52.2)
South-East Europe	91 (17.1)
North America	49 (9.2)
South America	9 (1.7)
Africa	4 (0.8)
Asia-Pacific	102 (19.1)
**Level of income**	
High-income	483 (90.6)
Middle-income	46 (8.6)
Low-income	4 (0.8)

Respondents were categorized into two main groups by occupation: HCWs (physicians, nurses) (249/533; 46.7%) and non-HCWs (other professionals) (284/533; 53.2%). “Other professionals” consisted of 152 office workers (28.5%), 64 employed in education and academia (12%) and 68 in other fields (12.8%). Gender and age distribution were comparable among HCWs and non-HCWs, but they differed with regard to the level of education, with a higher proportion of participants in the HCW group with a bachelor’s degree or higher (88.0% versus 78.2% in the other professions; p = 0.003). Responses from 49 countries were submitted. As shown in Table 1, the largest response was from Europe, followed by the Asia-Pacific region. Most respondents were from high-income countries.

### ILI and sickness presenteeism-related behavior

Most respondents (58.5%; 95% CI, 56.2–64.6) indicated that they would continue to work despite experiencing ILI symptoms. Analysis by occupational role showed no significant difference between HCWs (56.2%; 95% CI, 50.0–62.4) and non-HCWs (60.6%; 95% CI, 54.9–66.3) (p = 0.35). Staying at work while suddenly experiencing ILI symptoms was also common (65.7%; 95% CI, 61.6–69.7), with similar findings between HCWs (64.3%; 95% CI, 58.3–70.3) and non-HCWs (66.9%; 95% CI, 61.4–72.4) (p = 0.58). HCWs (45.8%; 95% CI, 39.6–52.0) were significantly less likely than other occupation groups (60.9%; 95% CI, 55.2–66.6) to avoid a colleague with ILI symptoms while at work (p = 0.001). There was no difference between HCWs (50.6%; 95% CI, 44.4–56.8) and non-HCWs (44.4%; 95% CI, 38.6–50.2) (p = 0.17) concerning avoidance behavior of colleagues presenting sick with ILI symptoms at work and wearing a surgical face mask. Similarly, avoidance behavior related to people with ILI symptoms outside of work during social encounters did not differ between HCWs (60.2%; 95% CI, 54.1–66.3) and other occupation groups (60.6%; 95% CI, 54.9–66.3) (Table 2).

**Table 2:** Responses to survey questions on influenza-like illness-related behavior by occupational group (healthcare workers and non-healthcare workers).

		No. (%) responding ‘yes’			
ILI-related behavior	No. responding	All	HCWs	Non-HCWs	*p* value^a^
I am willing to get the influenza vaccine	533	363 (68.1)	202 (81.1)	161 (56.7)	<0.001
I would avoid a person with ILI symptoms at social encounters	533	322 (60.4)	150 (60.2)	172 (60.6)	0.66
I would avoid a colleague with ILI symptoms at work	533	287 (53.8)	114 (45.8)	173 (60.9)	<0.001
I would avoid a colleague with ILI symptoms at work if they are wearing a surgical face mask	533	252 (47.3)	126 (50.6)	126 (44.4)	0.17
If I am at home and suddenly feel unwell with ILI symptoms, I would still go to work	533	312 (58.5)	140 (56.2)	172 (60.6)	0.35
If I am at work and suddenly feel unwell with ILI symptoms, I would still stay at work	533	350 (65.7)	160 (64.3)	190 (66.9)	0.58
After being sick with ILI, I returned to work feeling symptomatic^b^	289	182 (62.9)	83 (64.3)	99 (61.8)	0.75

^a^Calculated using the chi-square test of significance.

^b^Participants who indicated to have been sick with influenza in the previous two years. HCWs, healthcare workers; ILI, influenza-like illness.

Two-hundred eighty-nine (54.2%) respondents indicated that they had experienced an ILI during the previous two years and most had stayed home for 2–3 days during the illness period (46% HCWs; 45.5% non-HCWs; mean, 3.2 versus 2.9 days, respectively). Among these, most HCWs (64.3%; 95% CI, 56.0–72.6) and non-HCWs (61.8%; 95% CI, 54.3–69.3) returned to work feeling symptomatic, but no statistically significant differences were observed among the two categories (Table 2). Of note, HCWs (81.1%, 95% CI, 76.2–86.0) were significantly more willing to get the influenza vaccine compared to professionals in other occupations (56.7%; 95% CI, 50.9–62.5) (p<0.01).

### Sickness presenteeism related to the severity of symptoms

**Fig 1:**
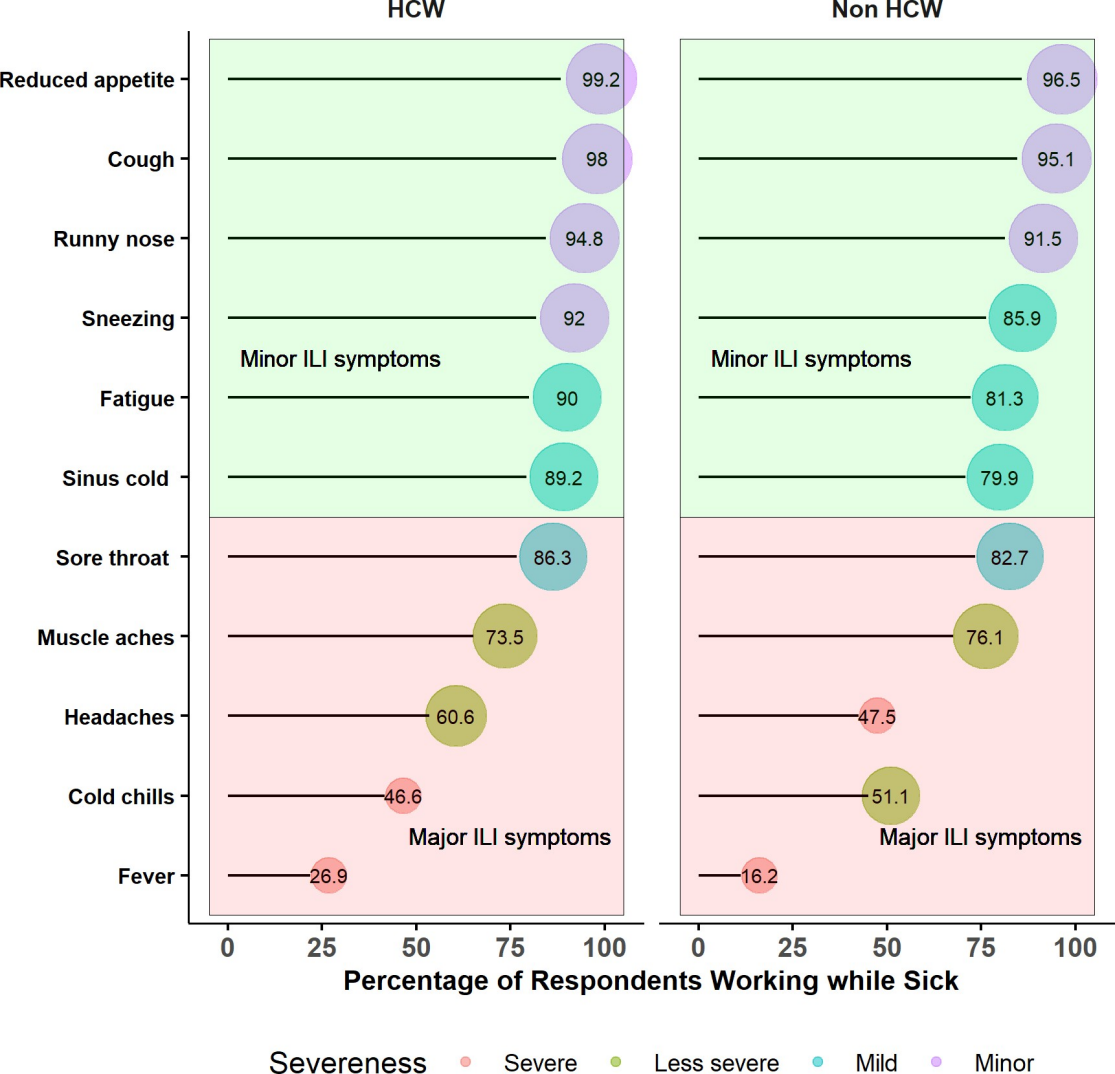
Sickness presenteeism related to the severity of influenza-like illness and influenza-like symptoms by occupational group. Pink: ILI symptoms classified as “major”. Green: ILI symptoms classified as “minor”. The radius of circles represents the percentage of respondents who would go to work with any of the symptoms. The bigger the circle, the higher the percentage of respondents reporting a symptom.HCW, healthcare worker; ILI, influenza-like illness.

Participants’ attitudes to sickness presenteeism while with ILI symptoms varied according to the severity of symptoms. Sixty-seven (26.9%) HCWs and forty-six (16.2%) non-HCWs responded that they would report to work with fever only (p<0 .01). HCWs and non-HCWs responded that they would continue to go to work with cold chills only (46.6% versus 51.1), headache (60.6% versus 47.5%), muscle aches (73.5% versus 76.1%) and sore throat (86.3% versus 82.7%). Almost all HCWs (89.2–99.2%) and non-HCWs (80–96.5%) affirmed that they would go to work with non-febrile “minor” ILI respiratory symptoms, including sore throat, sinus cold, fatigue, sneezing, runny nose, cough and reduced appetite (Fig 1).

### Sickness absenteeism related to a combination of symptoms

**Fig 2:**
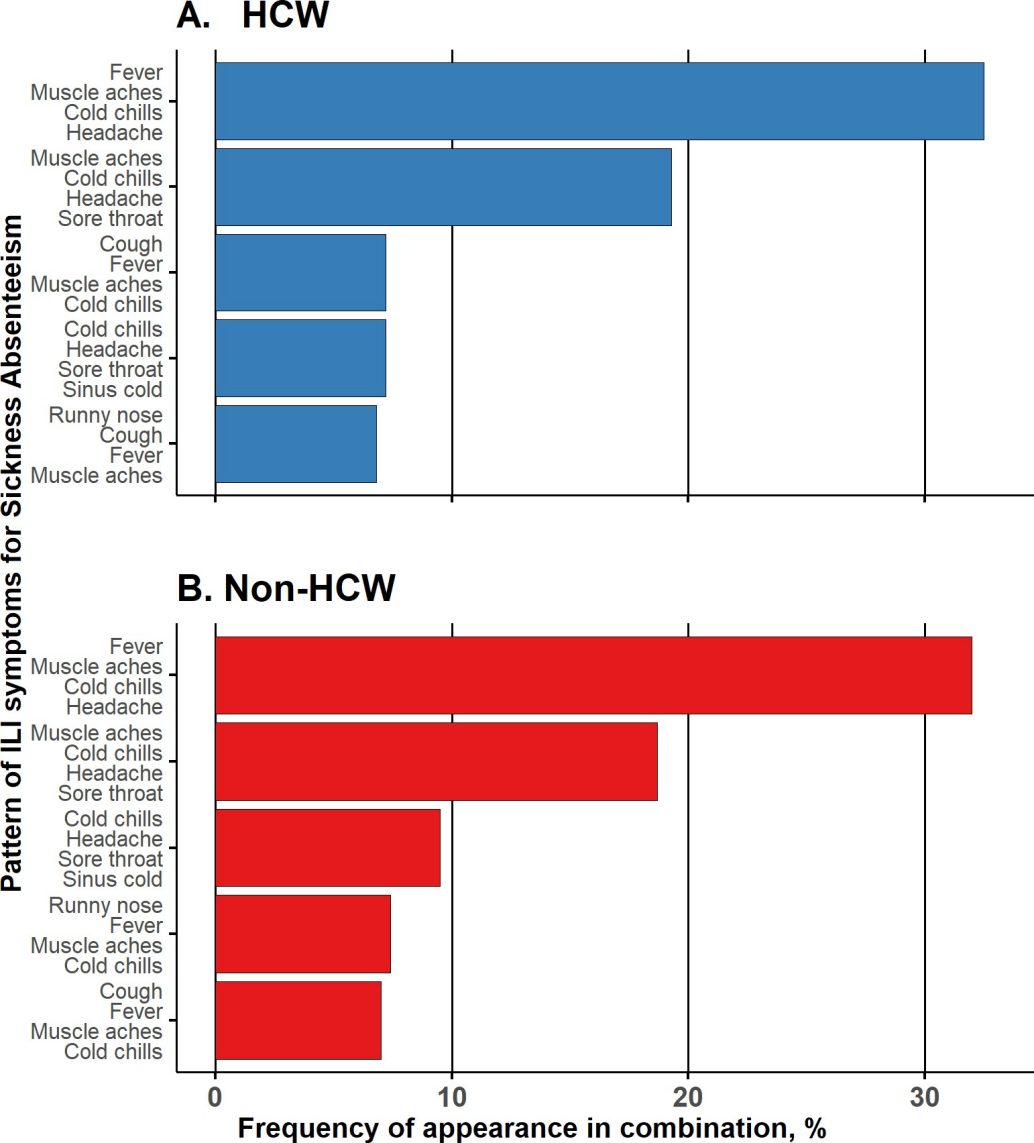
Combination of influenza-like illness and influenza-like illness symptoms reported for sickness absenteeism by occupational group. HCW, healthcare worker.

Using a “check all that apply” format for a list of 11 ILI symptoms, we further assessed the most unique and common patterns of ILI symptoms associated with sickness absenteeism, i.e. respondents staying at home away from work. Heterogeneity was observed among the patterns of ILI symptoms reported by HCWs and non-HCWs. The diversity of ILI symptom patterns was mirrored by the total number of 197 unique patterns reported (52 common patterns for both categories; 70 unique patterns for HCWs and 75 for non-HCWs). Fig 2 shows the distribution of the most frequently identified four ILI symptom patterns with which respondents would stay at home through illness. Analysis of the patterns revealed that the most prevalent ILI symptom combination (>30%) for both categories included fever, muscle aches, cold chills and headache (81 [32.5%] HCWs versus 91 [32%] non-HCWs), followed by sore throat, muscle aches, cold chills and headache as the second most common pattern (48 [19.3%] HCWs versus 53 [18.7%] non-HCWs) (Fig 2).

### Most relevant ILI symptoms

**Fig 3:**
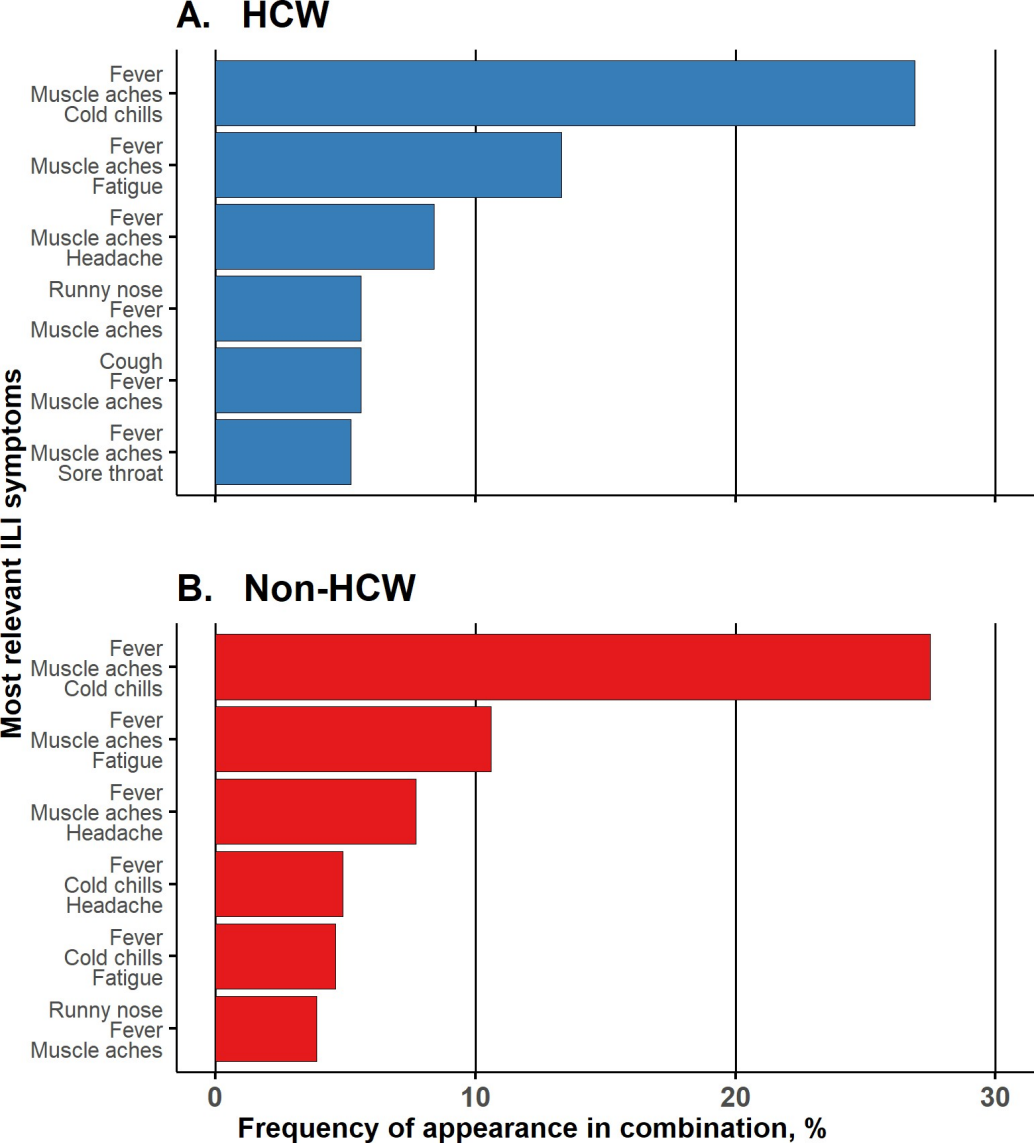
The most relevant influenza-like illness symptoms reported by occupational group. HCW, healthcare worker.

Analysis of the three symptoms considered by respondents as the most relevant for ILI revealed 82 total unique patterns (34 common patterns for both categories; 15 unique patterns for HCWs and 33 for non-HCWs) (Fig 3). The most commonly identified pattern by both HCWs and non-HCWs included fever, muscle aches and cold chills (67 [27%] and 78 [27.5%], respectively). Fever was included in all frequently identified three symptom combinations by HCWs and non-HCWs. The reasons that respondents felt would keep them at home through illness differed according to what they considered to be the most relevant ILI symptoms. Less than half of respondents selected the identical three symptoms when asked which are the most relevant ILI symptoms and with what symptoms they would stay away from work (120 [48.2%] HCWs and 139 [48.9%] non-HCWs).

## Discussion

We investigated the frequency of sickness presenteeism behavior among HCWs and other professionals, particularly ILI symptoms experienced while continuing to work through illness. Among our study respondents, working despite ILI illness was as prevalent in HCWs as in non-HCWs. Overall, 66% respondents reported staying at work while suddenly feeling unwell and 59% would come to work if feeling unwell while at home, even if suffering from several ILI symptoms. Our study adds to the growing body of literature highlighting the high level of employee sickness presenteeism, which has been reported previously in cross-sectional surveys as ranging from 40% to 90% [14–20; 22–26, 28]. Most participants reported having had an ILI during the previous two years. We found many similarities between the two categories, suggesting that certain behaviors and attitudes are prevalent among a range of occupational groups and persist in the healthcare environment where an awareness of patient vulnerability due to comorbidities should be of concern [14, 18, 22].

Our results suggest that most HCWs and non-HCWs would continue working despite “minor” ILI, based on their individual judgement rather than objective criteria presenting an unnecessary risk to patient safety and public health. However, it is not always easy for employees to decide when to stay away from work [20]. Of note, in a USA survey, a hospital sick leave policy asked febrile HCWs to refrain from working, but not those with respiratory symptoms in the absence of fever [25]. The risk of influenza transmission to patients from afebrile employees with influenza has been documented. Ridgway et al reported that 41% of HCWs with confirmed influenza were afebrile before their diagnosis [25]. The US Centers for Disease Control and Prevention recommend that HCWs with fever and respiratory symptoms do not work or should at least stop patient care activities until they are afebrile for 24 hours [27]. More than 80% of our survey respondents did not follow this recommendation.

Despite international guidelines [27], we note with concern that HCWs and non-HCWs would continue working despite “major” ILI symptoms, such as fever, cold chills, muscle aches and headache. More HCWs (27%) than non-HCWs (16%) would work with fever alone. Our findings also mirror research from the USA where a recent study reported that 33% worked with ILI symptoms, including fever, myalgia and cough, and 13% worked despite having a fever >38.5°C [17]. In another recent study, a higher rate was reported, and 35% respondents would work with fever alone [28]. This is a worrisome behavioral trend which can compromise quality of care and patient, HCW and coworker safety. Similar to other findings, HCWs frequently work while being sick. Suggested reasons for this may be linked to a sense of obligation to colleagues [9, 14, 15], organization, logistic and cultural factors [24, 29]. When HCWs are sick with ILI symptoms, they should not provide direct patient care.

In a study from New Zealand, the authors reported that more than one-third of HCWs surveyed perceived themselves as “not unwell enough” to stay home when sick. [26]. One of the pressures encountered by staff was the number of paid sick leave days allocated annually and those who had exhausted their allocation were unable to take leave when sick with ILI [26]. While some employees get paid sick leave days, others do not have this privilege and have no alternative but to work through illness [29]. Sickness presenteeism pressures differ at the national and local level in society according to social norms and economic income. Due to limited resources, many countries do not provide paid sick leave for employees with mild, unspecified respiratory symptoms [4,29]. This highlights the fact that the decision to work through illness is a complex phenomenon and is shaped not only by personal factors or lack of knowledge regarding the importance of the risk of transmission but, more importantly, it is inherent in the organizational culture and policies with financial penalties, which may place possible constraints on absenteeism, thus resulting in presenteeism [24]. Hence, treating people equally by addressing nonpunitive sick leave in various societal backgrounds is necessary.

Most countries that participated in this study are high-income nations (90%) from the European region (70%). Further exploration of sickness presenteeism across different regions, economic income levels and welfare systems is needed, mainly as the majority of studies have been conducted in high-income countries. Research is particularly needed in low-income settings where insufficient staffing levels are prevalent.

When participants were asked with which combination of ILI symptoms they would stay home when sick and which symptoms they considered as most relevant, 197 unique patterns were reported. Of note, responses differed between the two questions, suggesting a lack of clarity in what constitutes severe and mild ILI symptoms. A study by Szymczak et al reported a similar finding in that 57% of respondents perceived a certain ambiguity about what symptoms constitute being “too sick to work” [9]. This suggests that it is important to define the severity of ILI symptoms that should preclude employees from attending work.

Sickness presenteeism in the non-HCW category was also reported to be high. In a survey of school employees, 77% of individuals with ILI symptoms attended work through illness [16]. One of the most common factors for attending work with ILI symptoms was the perception that the individual felt not to be infectious [17]. Targeting educational messages to employees that address these gaps may be beneficial. Unfortunately, infection control guidelines specific to the non-healthcare work environment do not exist. Thus, pragmatic policies that consider the effective prevention of ILI and improved hygiene measures for employees is warranted.

HCWs were significantly more willing to receive the influenza vaccine compared to non-HCWs, although vaccination coverage among individuals with chronic diseases and HCWs is lower than 40% in most countries [27, 30] and its effectiveness varies each year [31]. This finding may stem from the fact that many healthcare facilities have widely endorsed influenza vaccination programs aimed at reducing influenza-associated complications and promoting patient safety. However, low- and middle-income countries are still at a disadvantage in terms of offering influenza vaccination for HCWs [27]. Health departments need to update their immunization policies and HCWs should be considered a priority for influenza vaccination. By doing so, leverage can be exerted on hospitals to encourage and provide their staff members with the influenza vaccine.

Of note, it is interesting that the avoidance of a colleague who presents at work with ILI symptoms varied considerably and non-HCWs were significantly more likely to show avoidance behavior. This may be explained by the chosen profession of HCWs. Being in contact with patients suffering of respiratory viruses, HCWs are often exposed to an increased risk of infection, whereas non-HCWs generally work with healthy individuals. Further, we found no significant difference in avoidance behavior of colleagues presenting sick at work and wearing a surgical face mask between HCWs and non-HCWs, suggesting a sense of reassurance from the protection of sick persons with a face mask.

Effective infection prevention measures including hand hygiene, promoting cough etiquette and the supply of face masks, have not yet been adequately addressed. For example, HCWs in many Asian countries wear cloth face masks or are required to buy their own face masks from local markets. These products are of inferior quality and put staff members at risk from influenza and other respiratory infections [32]. To prevent the transmission of ILI and other respiratory infections, such as the currently ongoing COVID-19 pandemic, healthcare facilities should consider stringent infection prevention hygiene measures for HCWs presenting with any respiratory symptoms and fever while providing clear guidance.

Organizations such as the World Health Organization, US Centers for Disease Control and Prevention and other country agencies need to invest more time in improving working conditions, including adequate staffing. Policy makers should act towards reducing sickness presenteeism behaviour, for example, by implementing policies that support sick leave days, that encourage employees to stay home when sick and that consider substitute employees. Most importantly, HCWs behavior and attitudes towards sickness presenteeism has wide-ranging implications for patient safety and the quality of health systems overall and strong action should be taken to nip the problem in the bud. Together with initiatives to provide high-quality health care, the pursuit of preventing healthcare-associated infections hinges upon improving both infection prevention programs and administrative systems worldwide to reduce presenteeism. Institutions also need to provide adequate resources, such as temporary health workers.

### Limitations

Our study has some limitations. First, our sample does not represent a random sample and, by the nature of its design, it is biased towards internet/social media users and people who are willing to complete surveys. Therefore, the generalizability of our results may have been affected. Second, the survey was distributed through the ISAC-IPC Working and results may reflect sampling bias. Third, the online invitation to complete the survey sent via social media platforms was seen by an unknown number of individuals leading to a lack of denominator data to the response rate. Fourth, study participants self-reported ILI symptoms, which may be subject to recall bias. Fifth, the number of respondents from other world regions, particularly low- and middle-income countries, was too small to permit meaningful comparisons between the two categories, which is an analysis that could provide valuable insight. Although drawn from a nonrepresentative sample of participants, the wide representation of HCWs and non-HCWs and representativeness among age/gender demographics indicate a meaningful range of responses and the general behavioral intentions towards sickness presenteeism in the population.

## Conclusions

Sickness presenteeism appears to be common among HCWs and other professionals and a concerted series of actions are needed to prevent the transmission of ILI and optimize safety. Single-measure approaches may be insufficient to interrupt ILI transmission and other multimodal intervention strategies should be considered, such as hand hygiene, isolation, appropriate use of personal protective equipment, the uptake of influenza vaccine, and discouraging presenteeism. A cultural change is required, as well as appropriate access to sufficient sick leave while experiencing ILI symptoms.

## Supporting information

S1 File (PDF)

S1 Data (XLSX)
