# Supplementary material for: Not sick enough to worry? "Influenza-like" symptoms and work-related behavior among healthcare workers and other professionals: Results of a global survey
Source: PLoS One. 2020 May 13;15(5):e0232168. doi: 10.1371/journal.pone.0232168 (PMC7219706; doi:10.1371/journal.pone.0232168)
Supplement: S1 File — (PDF) [file pone.0232168.s001.pdf]

Influenza / the flu

*International Society of Antimicrobial Chemotherapy
http://www.ischemo.org/welcome

Dear colleague / Dear friend,

Following last year’s extensive influenza season, the ISAC* infection prevention and control working group has launched this influenza survey, the purpose of which is to explore views and behaviour of the general public in the face of influenza.

The survey is anonymous and takes less than 5 minutes to complete. Your responses will be summarized along with other responses and used collectively to help guide recommendations in remaining gaps.

Feel free to share this link with your colleagues and friends in order to achieve a better population mix.

Your input would be very valuable.

On behalf of our working group, thank you for your support!

Prof. Dr. Andreas Voss
Radboudumc & CWZ, Nijmegen, The Netherlands
Chair ISAC-IPC working group

If you have any questions about this work, you can contact Katja.Saris-deLeuw@Radboudumc.nl

* 1. Please select your answer:

- ◯ I am 18 years of age or older and particpiate voluntarily
- ◯ I am younger than 18 years and/or I do not want to participate

Influenza / the flu

Questions about you

2. What is your gender?

- Female
- Male
- Other/Prefer not to say

3. What is your age?

- 18-19 years old
- 20-29 years old
- 30-39 years old
- 40-49 years old
- 50-59 years old
- 60-69 years old
- 70-79 years old
- 80-89 years old
- 90 years or older

4. In which country do you live (please write in full)?

5. Please select your highest degree or level of education you have completed:

- No education completed
- Primary / Elementary education
- High school / Secondary school education
- Lower or intermediate vocational education
- Higher technical/ vocational education
- Bachelor’s degree
- Master’s degree
- Doctorate degree
- I do not wish to answer

* 6. Please select the occupation that best fits your current status/job/internship/volunteer work (for example: I am a receptionist at a hospital, I will choose the answer “In a reception area”). I work:

- In education (primary, secondary, university level)
- In healthcare (with patient or client contact e.a. hospital, nursing home)
- In daycare (e.a. children), shelter (e.a. homeless/refugees)
- For the government (police, military, fire station)
- In transportation (bus, taxi, train, lorry)
- In retail/catering (with customer contact)
- In construction (construction, renovation, maintenance, installation)
- Mainly outdoors (farmer, gardener, road worker)
- In a reception area (front-office, back-office)
- At an office (in a company, organization, institution, Healthcare)
- I am a student
- I currently do not work/I am retired
- Other

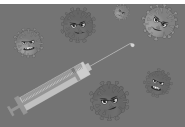

Influenza / the flu

Questions about influenza (or the flu)

7. Would you stay at home (away from work) if you were experiencing any of the following symptoms?

	No	Yes
Runny nose	○	○
Mild dry cough	○	○
Fever	○	○
Muscle aches	○	○
Cold chills	○	○
Pounding headache	○	○
Sore throat	○	○
Sinus cold	○	○
Reduced appetite	○	○
Fatigue	○	○
Sneezing	○	○

8. Would you stay at home (away from work) if you were experiencing a combination of any of the following symptoms?
Please tick the relevant boxes to create the combination of symptoms:

- ☐ Runny nose
- ☐ Mild dry cough
- ☐ Fever
- ☐ Muscle aches
- ☐ Cold chills
- ☐ Pounding headache
- ☐ Sore throat
- ☐ Sinus cold
- ☐ Reduced appetite
- ☐ Fatigue
- ☐ Sneezing

9. What do you consider the 3 most relevant flu symptoms? Please tick three symptoms below:

- ☐ Runny nose
- ☐ Mild dry cough
- ☐ Fever
- ☐ Muscle aches
- ☐ Cold chills
- ☐ Pounding headache
- ☐ Sore throat
- ☐ Sinus cold
- ☐ Reduced appetite
- ☐ Fatigue
- ☐ Sneezing

10. In your personal life would you avoid (stay away from) a person sick with flu/flu-like symptoms: fever, cold chills, pounding headache, muscle ache, coughing, fatigue?

- ○ No, I do not avoid (because it is impolite to do so or I do not think about it).
- ○ No, because I will wear a mask and practice hand hygiene.
- ○ No, because that is not possible.
- ○ Yes, personally I will always avoid a sick person.
- ○ Yes, when possible.

11. At your place of work would you avoid (stay away from) a person sick with flu/flu-like symptoms: fever, cold chills, pounding headache, muscle ache, coughing, fatigue?

- ○ No, I do not avoid (because it is impolite to do so or I do not think about it).
- ○ No, because I will wear a mask and practice hand hygiene.
- ○ No, because that is not possible.
- ○ Yes, I will always avoid sick colleagues.
- ○ Yes, when possible.

12. Would you avoid (stay away from) a person sick with flu/flu-like symptoms at your place of work, if they wear a face mask?

- ○ No, I do not avoid (because it is impolite to do so or I do not think about it).
- ○ No, because that is not possible.
- ○ Yes, I will avoid even if they wear a face mask.
- ○ Yes, when possible.

13. Would you get a flu vaccination/flu shot?

- ○ Yes, definitely (even if I have to pay for it myself).
- ○ Yes, but only when it is offered to me free of charge.
- ○ Maybe/I don’t know.
- ○ No.
- ○ No opinion.

14. Imagine the following scenario: You are at home and experience some mild flu-like symptoms (coughing, fatigue, headache, cold chills) and you feel that you are possibly getting sick with the flu. Would you go to work?
Please choose the answer that best represents your opinion:

- Yes, definitely.
- Yes, if the symptoms get worse I can always go home.
- No, in that case I will stay at home.

15. Looking back at the past 2 years, when you were sick with flu like symptoms, how many days did you stay at home, on average?

- 1 day
- 2-3 days
- 4-5 days
- 6-7 days
- 8-9 days
- Over 9 days
- I have not had the flu (in the past 2 years)

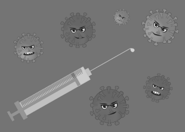

Influenza / the flu

Last page

16. When you returned to work after having been sick with the flu, how did you feel?

- I had some symptoms (coughing, sneezing, fatigue) and a fever, but I was capable of working.
- I had some symptoms (coughing, sneezing, fatigue), but I did not have a fever and I felt healthy enough to work.
- I did not have any symptoms, but not fully recovered.
- I did not have any symptoms and was fully recovered.

17. Imagine the following scenario: You are currently working and experience some mild flu-like symptoms (coughing, fatigue, headache, cold chills) and you feel that you are possibly getting sick. What would you do? Please select the answer that suits you best.

- I would stay at work no matter what.
- I would stay at work until the symptoms are so bad that I cannot do my job properly anymore.
- I would report sick and go straight home.

Thank you for participating.
